# ZFP57 promotes ovarian cancer progression by transcriptionally regulating BRCA1 and managing G1 checkpoint

**DOI:** 10.7150/jca.84601

**Published:** 2023-07-09

**Authors:** Weirong Fan, Rui Xiong, Ziyang Zhou, Cancan Zhang, Yanli Han, Tingyan Shi, Jianping Qiu, Rong Zhang

**Affiliations:** 1 The Third School of Clinical Medicine, Southern Medical University, Guangzhou, China.; 2 Department of Obstetrics and Gynecology, Fengxian Hospital Affiliated to the Southern Medical University, Shanghai, China.; 3 Department of Gynecologic Oncology, Zhongshan Hospital, Fudan University, Shanghai, China.; 4 The Affiliated Suzhou Hospital of Nanjing Medical University, Suzhou Municipal Hospital, Suzhou, China.

**Keywords:** Ovarian cancer, ZFP57, BRCA1, G1 checkpoint, Biomarker

## Abstract

Ovarian cancer (OC) which is one of the frequently-occurring gynecologic malignant tumors, endangers the health of women. The zinc finger protein 57 (ZFP57) plays crucial functions during the progression of cancer and is reported as a prognostic and therapeutic candidate in a variety of cancer. However, the biological function as well as the underlying mechanism of ZFP57 during OC progression remains unknown. Here, ZFP57 expression was found prominently increased in OC tissues and correlated with the prognosis of OC patients. Knock down of ZFP57 in OC cells inhibited the cell proliferation and migration, and also arrested the cells at G1 phase as well as accelerated the apoptosis. Additionally, ZFP57 transcriptionally regulated BRCA1 expression in OC, indicating that ZFP57 may affect BRCA1 mediated G1 checkpoint to regulate the cell cycle of OC cells and further influence the progression of OC. Taken together, our present study discovered a novel function of ZFP57 in OC, suggesting that ZFP57 could be potentially treated as a prognostic biomarker and therapeutic target for OC patients.

## Introduction

Ovarian cancer (OC) is a common gynecological malignancy, among which epithelial ovarian cancer nearly accounts for 90%. The mortality of OC ranks at the top of gynecological malignancies, because of high recurrence, high metastasis, and poor survival rate 1. Given that the ovary is located deep in the pelvic cavity and the symptoms of OC are insidious at an early stage, it is estimated that approximately 70% of patients with ovarian cancer are already advanced at first-diagnosis, thereby losing the best opportunity for treatment 2. It is reported that the five-year survival rate of advanced ovarian cancer is only 20%-30%, if OC patients can be diagnosed and treated early, the five-year survival rate of them could rise to 90%, thus early diagnosis and early clinical intervention for OC patients are very important 3, 4. Traditional diagnostic approach of OC relies mainly on medical imagology, which with low spatial resolution and valuable for evaluation of ovarian epithelial malignancies that are early stage or when lesions are small 5. At present, with the rapid development of molecular biology, an increasing number of tumor molecular markers have been found, which were closely related to the biological behavior and were involved in the occurrence and development of OC 2, 6.

The zinc finger protein 57 (ZFP57) gene is a transcriptional regulator, which maps to chromosome 6. The ZFP57 protein is comprised of 516 amino acids, which contains a KRAB domain and 6 zinc finger structures 7. Its KRAB domain can bind to Trim 28 to form a transcriptional regulator complex, and then combine with the promoter region of targeting gene to maintain gene imprinting and transcription 8, 9. Recently, several studies have confirmed that ZFP57 plays important roles in tumor initiation, development and metastasis. Zheng et al. reported that ZFP57 regulated MEST mediated Wnt/β pathway and inhibited the proliferation of breast cancer cells 10. ZFP57 has also been previously reported to induce IGF2 expression and subsequently promote the growth of tumor cells 11. Also, it has shown that ZFP57 promoted colorectal cancer (CRC) hepatic metastasis and could influence the prognosis of CRC patients 12. But the expression level and function as well as the related mechanism of ZFP57 in OC is not well investigated yet.

In this study, we explored ZFP57 expression via several related approaches in OC, and found that ZFP57 expression was correlated with clinicopathological information as well as prognosis of OC patients. Moreover, we investigated the effects and possible mechanisms of ZFP57 in OC cells, in order to provide more reliable factual supports and molecular targets for OC diagnosis and treatment.

## Materials and Methods

### Data Mining

The *ZFP57* gene expression data for ovarian adenocarcinoma was downloaded from TCGA, which were processed by Broad Institute's TCGA work group. The gene expression data for normal ovarian was downloaded from GTEx (https://gtexportal.org).

Gene Expression Omnibus (GEO) datasets GSE14407 (https://www.ncbi.nlm.nih.gov/geo/query/acc.cgi?acc=GSE14407), GSE18520 (https://www.ncbi.nlm.nih.gov/geo/query/acc.cgi?acc=GSE18520), GSE40595 (https://www.ncbi.nlm.nih.gov/geo/query/acc.cgi?acc=GSE40595) were also applied in this study to analyze *ZFP57* gene expression in ovarian cancer.

Lu ovarian and Hendrix ovarian datasets deposited in Oncomine database (https://www.oncomine.org) were used. A combined filter was applied to display the corresponding datasets. The Cancer Type was defined as Ovarian Cancer and Data Type was mRNA, and Analysis Type was Cancer versus Normal Analysis. The expression levels of* ZFP57* gene were read from the displayed bar chart and these data were parsed into Excel to analyze.

Survival rate analyzed by a Kaplan-Meier analysis of Ovarian cancer patients were referenced from an online database-The Kaplan Meier plotter (https://kmplot.com/analysis/).

### Clinical samples

Samples including human ovarian cancer, ovary ovarian cysts as well as normal ovarian epithelium were all from Department of Obstetrics and Gynecology, Fengxian Hospital, Southern Medical University. Tissues were all from informed consent and all protocols were approved by the ethical review committee of the World Health Organization Collaborating Center for Research in Human Production. The inclusion criteria are as follows. All patients were diagnosed as EOC by pathological findings excluding ovarian germ cell tumors, sex cord stromal tumors, recurrent and metastatic tumors; All patients are initially diagnosed and treated, without a history of ovarian surgery, no history of other tumors, no history of radiotherapy and chemotherapy, no history of immunotherapy, no immune deficiency, and no serious complications of internal and external medicine; none of the patients had a history of other tumor or related anti-tumor therapies including radiotherapy and chemotherapy before surgery.

### Cell culture and Regents

Human OC cell lines CAOV3, SKOV3, OV429, OVCAR3 and OVCAR8 were all preserved in Shanghai Cancer Institute. All above cell lines were cultured in the indicated medium with 10% fetal bovine serum (FBS, Biological Industries (BI)) and 1% antibiotics in a humidified incubator with 5% CO_2_, 37 ℃. Negative for Mycoplasma contamination of all above cell lines were verified before them used for further assay.

### Construction of plasmids and cell transfection

OV429 and OVCAR3 cell lines were cultured overnight for 60-70% confluence, and then transfected either with si-ZFP57 or the non-targeted siRNA as a negative control. The siRNA oligonucleotides for ZFP57 were purcahsed from GenePharma (Shanghai, China). The siRNA sequences were shown as follows: Si-ZFP57-1, sense (5'-3'): GCUUGAGCAAGAGGAA, antisense (5'-3'): UUCCUCUUCUUGCUCAAGC; Si-ZFP57-2, sense (5'-3'): GAAAGAGCUUCGAGAACAA, antisense (5'-3'): UUGUUCUCGAAGCUCUUUC. Negative control was scrambled siRNA, which targets no known gene sequence. The transfection procedures were completed using Lipofectamine® RNAiMAX (Thermo Fisher Scientific, 13778030).

The ZFP57 plasmid (EX-L0457-Lv102) and control vector plasmid were purchased from GeneCopoeia (Guangzhou, China). The transfection procedures were performed using Lipofectamine 3000 (Thermo Fisher Scientific, L3000015) according to the manufacture's protocols.

### RNA-seq analysis

Total RNAs were isolated using Trizol reagent (Invitrogen, 15596026) from OV429 cells transfected with Si-ZPF57 or control siRNA. These RNAs were employed for RNA-Seq and subsequent analysis by Majorbio (Shanghai, China).

### Western Blotting (WB)

RIPA buffer (Beyotime, P0013B) with 1mM PMSF (Beyotime, ST506) and protease inhibitor cocktail (Bimake, B15001) was used to lysed EC cells. Then protein contained in EC cell lysis solution were divided using accurate SDS-PAGE gels. After transferring onto nitrocellulose membrane (Shanxi, China), the protein was blocked by 1%BSA within PBS/Tween-20 for 1 hour. Next, the NC membrane was further cultured with anti-ZFP57 (1:1000, Immunoway, YT7618), anti-BRCA1 (1:1000, Proteintech, 22362-1-AP), anti-β-Tubulin (1:10000, Proteintech, 66240-1-Ig) and anti-GAPDH (1:10000, Proteintech,10494-1-AP) overnight at 4 °C. After washing, the NC membranes were further incubated using corresponding secondary antibody and was used for detecting targeted protein signals.

### *In vitro* cell behavior assays

Cell proliferation assay: 1,500 cells/well was seed in a 96-well plate cultured for 24, 48, 72, 96 and 120 h. Cell Counting Kit-8 (CCK8) assay was performed to measure cell viability according to manufacturer's instructions (CCK-8, Share-Bio, China). In colony formation assay, 1000 cells/well was cultured in 2ml complete culture media for 14 days. Then the colonies were dyed with 0.1% crystal violet and counted.

Cell migration assay: 2.5x10^4^ cells were seeded into the upper chamber of the transwell plate (Millipore, USA) and then the migrated cells were fixed and stained with 0.1% crystal violet after migrating for 24h. For the quantification, 6 randomly selected regions were photographed, and the cell numbers was counted.

Wound healing assay: 1 × 10^6^ cells/well was cultured in 6-well plates with 95% confluent. Sterile 1 ml pipette tips were used to make wounds in the cell layer and then the cells were washed with PBS twice. Then, serum-free medium was added 2 days later, the scratch width was measured using the inverted fluorescence microscope (Zeiss, Oberkochen, Germany). The quantification was applied using ImageJ software.

### Cell cycle and cell apoptosis assay

Cell cycle assay: OC cell lines were washed with PBS, then fixed in ethanol for 2 hours at 4 °C. Washing with PBS for two times, and suspended in PBS containing RNase A and PI and incubated in darkness at RT for 15 mins. The different phases of the cells were measured using flow cytometry.

Cell apoptosis assay: Annexin V-FITC Kit (Thermo Fisher Scientific, #88800574) was used for this assay. OC cells were collected and incubated with Annexin V-FITC in the provided binding buffer for 15 min in the dark at RT. After washing, PI (Thermo Fisher Scientific, #BMS500PI) was added and analyze by flow cytometry. The data were analyzed using Flow Jo software v 10.4.2 (Flow Jo).

### Immunohistochemistry (IHC) staining

As described previously in literature, IHC staining was performed 13. In brief, the paraffin-embedded EC and normal tissue slides were deparaffinized and rehydrated, and then blocked using 10%BSA in PBS for 30 mins. Then, incubated overnight at 4 °C using ZFP57 antibody (Novus Biologicals, 1:100, NBP1-91550), and incubated using the matching secondary antibodies for 1 h at RT. Then DAB substrate kit (Thermo Scientific) was used for treating the sections and hematoxylin staining for nuclear counterstaining. Last, a microscope (Carl Zeiss) was used for photographed and analyzed all the sections.

### Construction of Plasmids, dual-luciferase reporter assay

BRCA1 promoter-luciferase reporter plasmids containing the promoter region were built in the pGL4.10/ pGL3B plasmid. Wild-type and mutants promoter luciferase constructs were proofed by DNA sequencing. A dual luciferase reporter assay (Promega, WI, USA) was performed as per manufacturer's user guide.

### Statistical analysis

Graph Pad Prism 9 version software (San Diego, CA) was used for statistical analyses. Data were expressed as the means ± SEM. Data comparisons between groups was performed by One-way ANOVA or two-tailed student's T-test. P<0.05 were perceived as statistically significant.

## Results

### ZFP57 is highly expressed and predicts poor prognosis in OC patients

**Figure 1:**
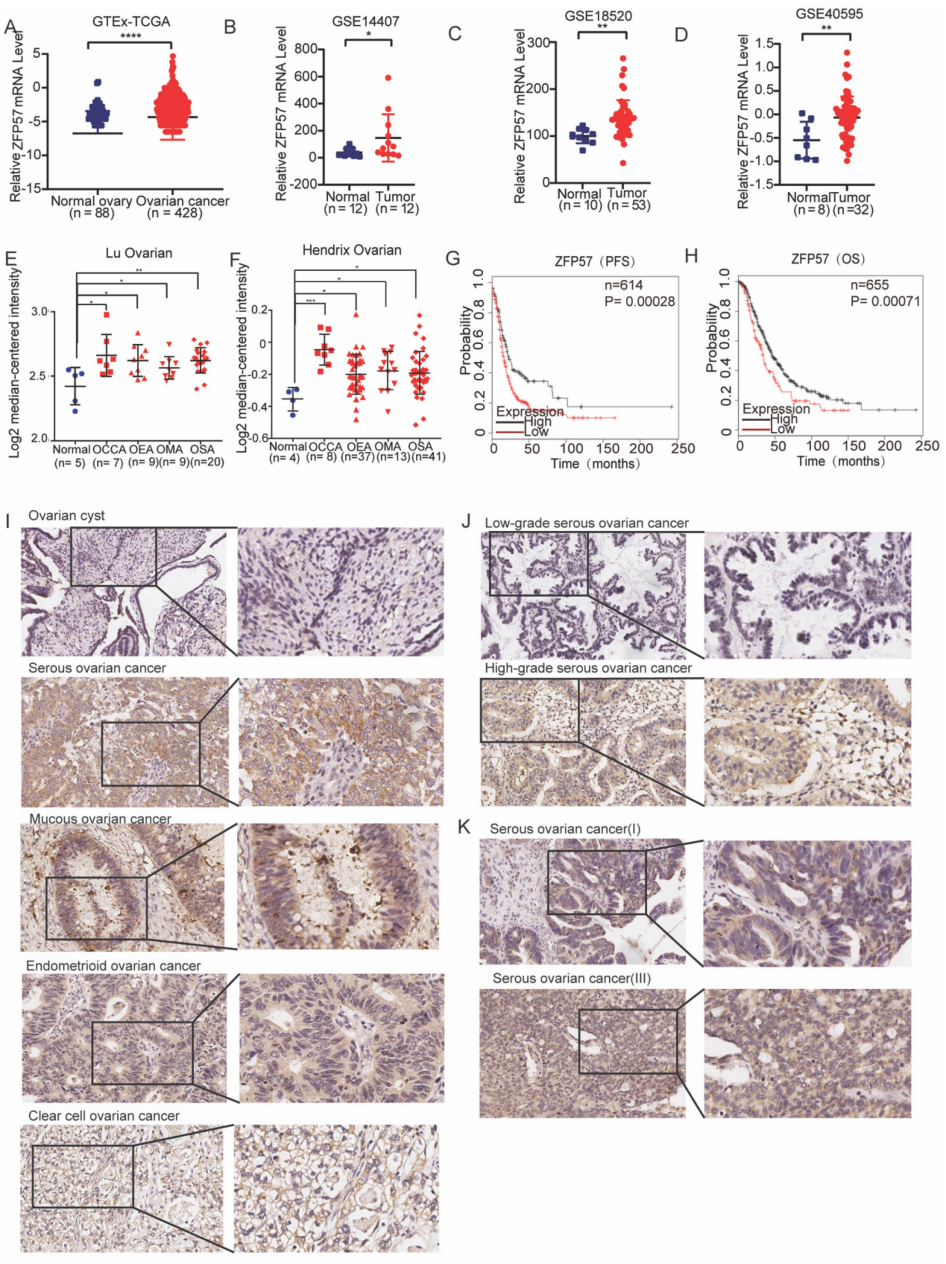
** ZFP57 is highly expressed in OC and predicts poor prognosis. A-F:*** ZFP57* expression in ovarian cnacers and normal tissues using TCGA, GTEx, Oncomine and GEO database. OSE: Ovarian Surface Epithelium; OCCA: Ovarian Clear Cell Adenocarcinoma; OEA: Ovarian Endometrioid Adenocarcinoma; OMA: Ovarian Mucinous Adenocarcinoma; OSA: Ovarian Serous Adenocarcinoma. **G-H:** Kaplan-Meier analysis of the correlations between ZFP57 expression and OS/PFS of OC patients based on an online database. **I.** Representative IHC images of ZFP57 in 51 ovarian cyst and 151 various ovarian cancer subtypes clinical samples. Scale bar is 50 μm. **J.** Representative IHC images of ZFP57 in high-grade ovarian cancer and low-grade ovarian cancer. Scale bar is 50 μm.** K.** Representative IHC images of ZFP57 in stage I and stage III ovarian cancer. Scale bar is 50 μm. Data are presented as means ± SEM. *P < 0.05; **P < 0.01; ***P < 0.001, ****P < 0.0001.

To investigate the function of ZFP57 in OC, we firstly analyzed the sequencing data of OC in TCGA and normal ovarian tissue from GTEx, and the results showed that the mRNA expression of ZFP57 in ovarian serous cystadenocarcinoma was increased significantly compared to normal ovarian tissue (P < 0.0001, Figure 1A). Then we analyzed 3 independent ovarian cancer microarray data (GSE14407, GSE18520, GSE40596) in the GEO database and found that ZFP57 mRNA expression was also increased significantly in OC tissues compared with normal ovarian surface epithelium tissues (all P values were less than 0.05; Figure 1B-D). We further analyzed the ZFP57 mRNA expression level in the Lu Ovarian and Hendrix Ovarian databases on the Oncomine website. The results showed that in the above two databases, the ZFP57expression in OC (primary ovarian tumors/serous ovarian tumors) was significantly higher than that in normal ovarian surface epithelium or ovary (all P values were less than 0.05, Figure 1E and F).

To analyze the associations between ZFP57 expression and the prognosis of OC patients, univariate overall survival (OS) and progression-free survival (PFS)prognostic analysis were performed based on high and low ZFP57 expression in the Kaplan Meier-plotter database. The results showed that the OS and PFS time of patients with high ZFP57 expression were significantly shorter than those of patients with low ZFP57 expression (all P values were less than 0.0001, Figure 1G-H), suggesting that high expression of ZFP57 was closely associated with the poor prognosis of patients with OC.

To further verify the above database analysis results, we used IHC to detect the expression of ZFP57 in 51 ovarian cyst and 151 various ovarian cancer subtypes clinical samples (123 serous ovarian cancer samples, 10 mucinous ovarian cancer samples, 11 endometrioid ovarian cancer samples and 7 clear cell ovarian cancer samples) and found that the ZFP57 expression in various types of OC was significantly increased compared to that in cysts (Table 1, Figure 1I). Further analysis of the relationship between ZFP57 and the clinicopathological features of OC, we found that the expression of ZFP57 in high-grade serous ovarian carcinoma (HGSOC) was significantly upregulated than that in low-grade serous ovarian carcinoma (LGSOC), and the ZFP57 expression in advanced serous ovarian carcinoma (SOC) was significantly higher compared to that in early SOC, which were statistically significant (Table 2, Figure 1J-K). These data together indicated that high expression of ZFP57 was closely related to high-grade and advanced SOC.

### ZFP57 promotes the cell proliferation and inhibits the cell apoptosis in OC cells

**Figure 2:**
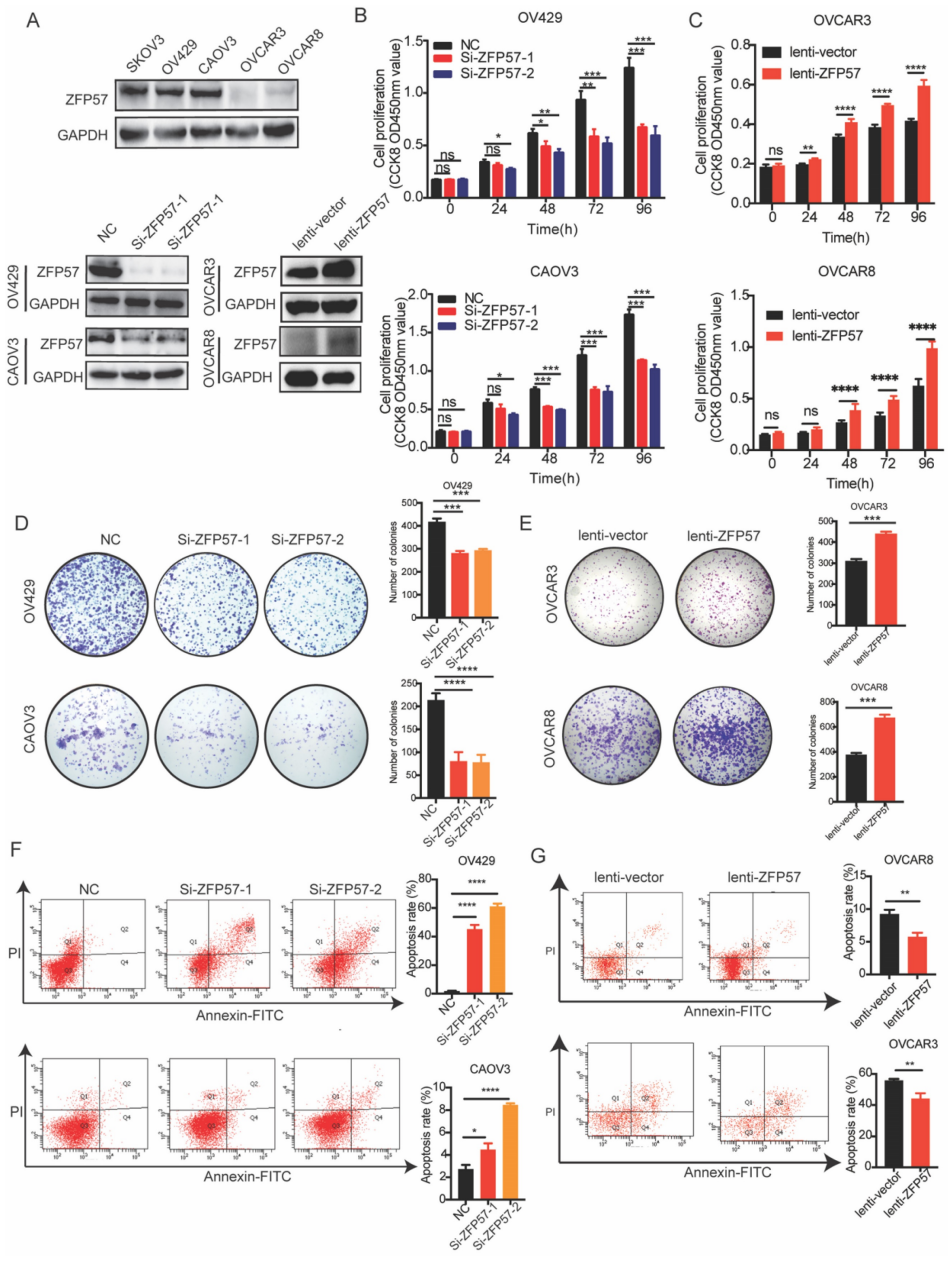
** ZFP57 promotes the proliferation and inhibits apoptosis of ovarian cancer cells. A.** Protein expression of ZFP57 in five OC cell lines. Interference efficiency verification of ZFP57 in CAOV3 and OV429 cells. Overexpression efficiency verification of ZFP57 in OVCAR3 and OVCAR8 cells. **B.** Relative cell viability of CAOV3 and OV429 cells expressing NC or Si-ZFP57. **C.** Relative cell viability of OVCAR3 and OVCAR8 expressing lenti-vector or lenti-ZFP57.** D-E.** Clone formation experiments of CAOV3 and OV429 cells expressing NC or Si-ZFP57 and OVCAR3 and OVCAR8 cells expressing lenti-vector or lenti-ZFP57.** F-G.** Flow cytometry for detection of apoptosis by Annexin/PI double staining in CAOV3 and OV429 cells expressing NC or Si-ZFP57 and OVCAR3 and OVCAR8 cells expressing lenti-vector or lenti-ZFP57. Data are presented as means ± SEM. *P < 0.05; **P < 0.01; ***P < 0.001, ****P < 0.0001.

To explore the biological functions of ZFP57 in OC, firstly we selected five OC cell lines (SKOV3, OV429, CAOV3, OVCAR3, OVCAR8), and used western blot experiments to detect the ZFP57 protein expression in these cell lines (Fig 2A). Then the specific ZFP57 interference experiments were performed on OC cell lines OV429 and CAOV3 with high expression of ZFP57, and ZFP57 overexpression experiments were performed on OC cell lines OVCAR3 and OVCAR8 with low expression of ZFP57.

After verifying the efficiency, we conducted a series of functional experiments. First, we used CCK8, clone formation and cell apoptosis experiments to examine the influence of ZFP57 on the proliferation and apoptosis of OC cells. CCK8 experiment showed that the proliferation ability of OV429 and CAOV3 with ZFP57 interference in OC cells was significantly inhibited (Figure 2B), and the proliferation ability of OVCAR3 and OVCAR8 with ZFP57 overexpression was significantly enhanced (Figure 2C). The clone formation assay showed that the clonogenic ability of OV429 and CAOV3 with ZFP57 interference was significantly inhibited (Figure 2D), and the clone formation ability of OVCAR3 and OVCAR8 with ZFP57 overexpression was significantly enhanced (Figure 2E). We also analyzed the effect of ZFP57 expression on OV429 and CAOV3 cell apoptosis by flow cytometry and found the apoptosis with ZFP57 interference was significantly increased (Figure 2F), and the apoptosis rate of OVCAR3 and OVCAR8 with ZFP57 overexpression was significantly inhibited (Figure 2G). Based on the above results, ZFP57 significantly promoted the cell proliferation and inhibit the cell apoptosis of OC cells.

### ZFP57 promotes the cell migration of OC cells

**Figure 3:**
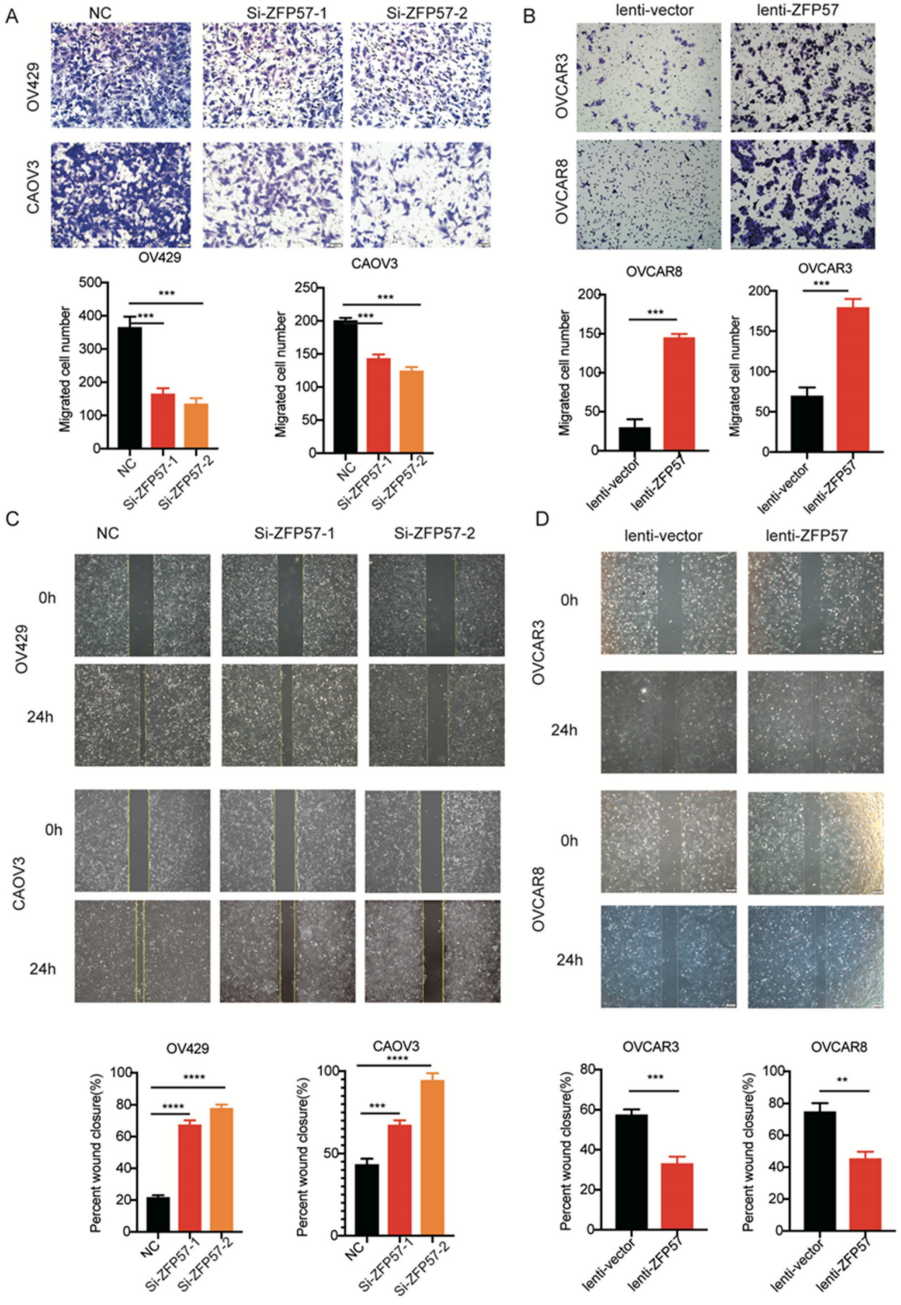
** ZFP57 promotes the cell migration of ovarian cancer cells. A.** Cellular migration ability was detected by transwell migration in CAOV3 and OV429 cells expressing NC or Si-ZFP57, representative pictures on the up and the number of migrated cells on the down. **B.** Cellular migration ability was detected by transwell migration in OVCAR3 and OVCAR8 expressing lenti-vector or lenti-ZFP57, representative pictures on the up and the number of migrated cells on the down.** C.** Cellular migration ability was detected by wound healing assay in CAOV3 and OV429 cells expressing NC or Si-ZFP57, representative pictures on the up and the percent wound closure on the down. **D.** Cellular migration ability was detected by transwell migration in OVCAR3 and OVCAR8 expressing lenti-vector or lenti-ZFP57, representative pictures on the up and percent wound closure on the down. Data are presented as means ± SEM. ***P < 0.001, ****P < 0.0001.

To investigate the effect of ZFP57 on the OC cells migration ability, the migration ability was tested using transwell assay. The results showed that migration ability of OV429 and CAOV3 with ZFP57 interference were significantly decreased and migration ability of OVCAR3 and OVCAR8 with ZFP57 overexpression were significantly increased (all P values < 0.001, Figure 3A-B).

We also tested the effect of ZFP57 on the migration ability using wound healing assay. The results showed again that the migration ability of OV429 and CAOV3 was significantly decreased after ZFP57 interference, the migration ability of OVCAR3 and OVCAR8 was significantly increased after ZFP57 overexpression (all P values < 0.001, Figure 3C-D). The above data indicated that high ZFP57expression in OC cells promoted the migration of OC cells.

### ZFP57 affects G1 phase arrest in OC cells

**Figure 4:**
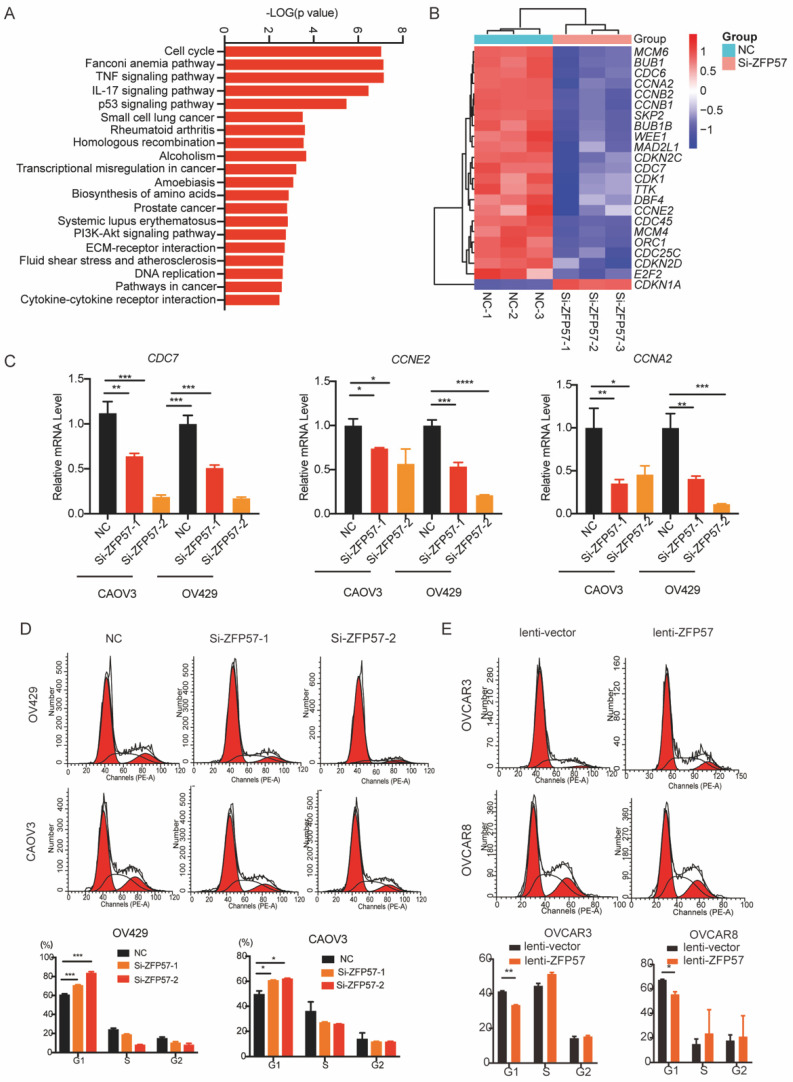
** ZFP57 affects G1 phase arrest in OC cells. A.** Top 20 KEGG pathways of target genes on the data from RNA-seq. **B.** Heat map of cell cycle related genes using our RNA-Seq data.** C.** The mRNA expression of *CDC7, CCNE2* and *CCNA2* in CAOV3 and OV429 cells expressing siNC and siZFP57. **D.** Cell cycle was detected by FACS analysis in CAOV3 and OV429 cells expressing NC or Si-ZFP57, representative pictures on the top and percent of phase on the button. **E.** Cell cycle was detected by FACS analysis in OVCAR3 and OVCAR8 expressing lenti-vector or lenti-ZFP57, representative pictures on the top and percent of phase on the button. Data are presented as means ± SEM. *P < 0.05; **P < 0.01; ***P < 0.001, ****P < 0.0001.

To explore the molecular mechanism of ZFP57 in OC, high-throughput transcriptome sequencing (RNA-seq) in OV429 cells with ZFP57 interference were performed. The results showed that specific interference ZFP57significantly affected the cell cycle of OC cells (Figure 4A). Meanwhile, the heatmap results of our cluster analysis showed that ZFP57 also significantly affected cell cycle-related genes, such as *CDKN1A, CCNA2, CCNE2, CDC7* so on (Figure 4B). Our RT-PCR results in OV429 and CAOV3 cells verified that the expression of *CCNA2, CCNE2, CDC7* were significantly reduced after ZFP57 interference (Figure 4C).

Then we applied the cell cycle analysis of OC cells using flow cytometry. Interfering with ZFP57 increased the percentage of OV429 and CAOV3 cells arrested in G1 phase and overexpression ZFP57 decreased the percentage of OVCAR3 and OVCAR8 cells arrested in G1 phase (Figure 4D and 4E). We thus concluded that ZFP57 acted to affects the G1 phase arrest in OC cells.

### ZFP57 transcriptionally regulates BRCA1 expression to affect the cell cycle of OC cells

**Figure 5:**
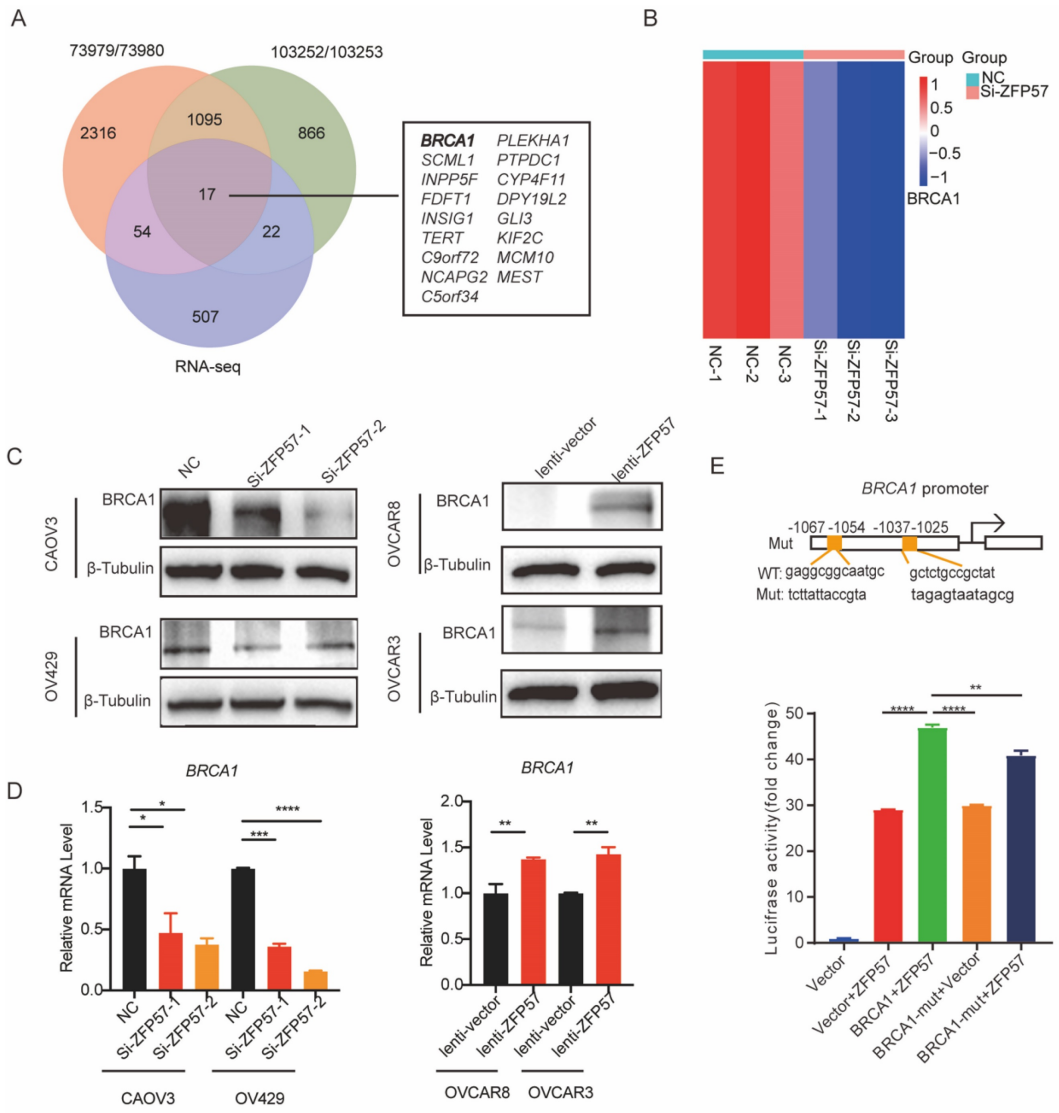
** ZFP57 transcriptionally regulated the expression of BRCA1 to regulate the cell cycle. A.** Intersection of RNA-seq and CHIP-seq target genes. ChIP-Seq data using Cistrome Data Browser (CistromeDB: 73979/ 73980, 293T cells; CistromeDB: 103252/ 103252, H1, Embryonic Stem Cell.) B, Heat map of BRCA1 using our RNA-Seq data.** C.** BRCA1 protein expression in CAOV3 and OV429 cells expressing NC or Si-ZFP57, and OVCAR3 and OVCAR8 expressing lenti-vector or lenti-ZFP57.** D**. *BRCA1* mRNA expression in CAOV3 and OV429 cells expressing NC or Si-ZFP57, and OVCAR3 and OVCAR8 expressing lenti-vector or lenti-ZFP57. **E.** The binding sites of ZFP57 to the promoter region of BRCA1 predicted by MEME and luciferase reporter assay was performed using 293T cells after transfecting the wild type plasmids and mutated plasmids (mutation site: orange). Data are presented as means ± SEM. *P < 0.05; **P < 0.01; ***P < 0.001, ****P < 0.0001.

To explore target genes transcriptional regulated by ZFP57 as a transcription factors, we analyzed Chromatin Immunoprecipitation Sequencing (ChIP-Seq) data using Cistrome Data Browser. Further synthesizing the sequencing results of RNA-seq, we found that Breast cancer gene 1 (BCRA1) may be a target gene of ZFP57. BRCA1 has been found the induction of G1 arrest by exogenous BRCA1 expression is likely to be associated with activation of p27^KIP1^ 14. To further explore the relationship between ZFP57 and BRCA1, we analyzed the results of RNA-seq and found that the expression of BRCA1 was significantly reduced after ZFP57 interference. We further studied the expression of BRCA1 using western blotting and RT-qPCR analyses. Interfering with ZFP57 led to the downregulation of BRCA1expression in OV429 and CAOV3 cells and overexpressing with ZFP57 led to upregulation of BRCA1 in OVCAR3 and OVCAR8 cells at both protein and mRNA levels (Figure 5C-D).

To further explore whether ZFP57 transcriptionally regulates BRCA1, we used MEME software to predict the binding site of ZFP57 to BRCA1 and found ZFP57 had binding sites with the BRCA1 promoter regions. Luciferase reporter assays further confirmed that the transcriptional activity of the BRCA1 promoter was significantly induced by ZFP57 and was significantly decreased by BRCA1 promoter mutations (Fig. 5E). These data suggest that ZFP57 can directly regulate the expression of BRCA1 at the transcriptional level in cells.

## Discussion

OC is one of the most serious malignancies affecting women's health, although conventional treatment strategies have improved the overall survival rate of OC patients, the fatality rate of OC is still stubbornly high due to its malignant phenotype characteristics such as invasion, metastasis and recurrence 3, 15. ZFP57 has a highly conserved structural sequence and participates in the neural development of vertebrates 16. ZFP57 gene is also involved in regulating the formation of neural crest in mice 17. In recent years, studies have suggested that high ZFP57 expression is associated with breast cancer, lung cancer, fibrosarcoma, and glioma, which could regulate Wnt/β-Catenin signaling pathway or IGF2, leading to the imbalance of expression of downstream oncogenes such as c-myc or phosphorylated AKT, further influencing the invasion and metastasis of tumor cells 10, 11, 18. It can also regulate the level of DNA methylation by interacting with DNA methyltransferase 1 (DNMT 1), DNMT 3A and DNMT 3B in tumors, and promote the abnormal hypermethylation of CpG island of tumor suppressor gene, thus promoting the occurrence and development of cancers 10, 19.

According to mRNA data downloaded from the TCGA/GTEx/GEO/Oncomine database, ZFP57 was significantly overexpressed in OC tissue compared to adjacent normal tissue, which was confirmed by our immunohistochemistry results. Bioinformation and immunohistochemistry analyses of ZFP57 are demonstrated from mRNA and protein levels respectively, although there is no direct comparability, the conclusions are consistent. The clinical pathological analysis in the table shows that the high expression of ZFP57 in ovarian cancer is related to benign and malignant, clinical staging, pathological grading of OC patients. Tumor stage have been reported to be important factors affecting the prognosis of ovarian cancer 20-23. Indeed, when compared with high-grade serous ovarian cancer, low-grade serous ovarian cancer is associated with improved survival in present study. Therefore, we speculate that ZFP57 may be a good prognostic marker of high-grade serous ovarian cancer.

At the same time, the results of cell function experiments showed that ZFP57 promoted the proliferation and migration of OC cells and inhibited their apoptosis, which may through affecting BRCA1 mediated G1 cell cycle regulatory point to regulate the cell cycle of OC cells and further influence the progression of OC. And high ZFP57 expression was closely correlate with poor prognosis of OC patients.

Programmed mitosis of tumor cells is a basic process in their life activities 24. The loss of G1 and G2 regulatory points induces tumor cells to enter the process of apoptosis, which is the beginning of malignant proliferation restriction of tumor cells 25. Tavares DC et al. showed that blocking tumor cells in G0/G1 phase by Guttiferone E (GE) was associated with increased apoptosis rate of melanoma cells 26. Park and his group have confirmed that relieving G2/M phase block can significantly dispel the inhibition of arsenic trioxide on the growth of lung cancer cells *in vivo* 27. Ghavami et al. showed that regulating the proportion of AGS gastric cancer cells in G1 and G2 phases can significantly reduce the resistance of cancer cells to cisplatin and other chemotherapy drugs, and further induce cell apoptosis 28. The results of our study showed that after interference of ZFP57, the proportion of OV429 and CAOV3 cells in G1 phase and their apoptosis rate was significantly increased, which indicated that inhibiting the expression of ZFP57 limited the mitotic process and accelerated the apoptosis rate of OC cells.

The results of ChIP-Seq and luciferase reporter assays showed that ZFP57 transcriptionally regulated BRCA1 gene, which played important roles in cell proliferation, differentiation, apoptosis and other life activities 29, 30. Mutation of BRCA1 is closely related to the occurrence, malignant invasion and metastasis of human tumors 31-33. It has been found that one of the most important biological functions of BRCA1 is to participate in the regulation of cell cycle 34. Disordered cell cycle will cause genomic instability, which will lead to the formation and malignant proliferation of tumor cells 35. In addition, deactivation of BRCA1 could induce P27 mediated cell cycle arrest 14, 36. Thus, ZFP57 may through transcriptionally regulate the increase or decrease of BRCA1 level to modulate the cell cycle, proliferation and apoptosis of OC cells, and further affect the process of OC.

Zinc finger protein members are the largest transcription factor family and have been studied in tumors. Together with these studies, ZFP57 could suppress breast cancer and promote liver metastasis in rectal cancer, indicating the cancer-specific effects and diversity of functional mechanisms of ZFP57, which deserves our attention and in-depth research. Our research found that expression of ZFP57 was significantly increased in OC and associated with poor prognosis. BRCA1 has a relationship within breast cancer and OC and cell cycle is very important to any types of cells, our study revealed a previous unprecedented mechanism that ZFP57 accelerates cell cycle by regulating BRCA1 and G1 checkpoint. Therefore, our investigations broaden the knowledges regarding the expression pattern, oncogenic roles, and cellular mechanism of ZFP57 in human cancers.

In summary, highly expressed of ZFP57 in ovarian cancer was significantly correlated with advanced SOC and high-grade OC, and also indicated poor prognosis of OC patients. After inhibiting the expression of ZFP57, the proliferation and migration of OC cells was suppressed. In addition, ZFP57 together with BRCA1 could manage the G1 cell cycle regulatory point and further affect the apoptosis of OC cells. The discovery of abnormal expression and tumor related function of ZFP57 in OC indicated it may become a new diagnosis-related biomarker and a therapeutic protein in the treatment of OC.

## Figures and Tables

**Table 1:** Relationship between ZFP57 expression and clinical pathological parameters of patients in epithelial ovarian cancer.

		ZFP57 High	ZFP57 Low	Total	χ^2^	P
	Epithelial OC	82 (54.3%)	69 (45.7%)	151	25.470	<0.001
	Ovarian cyst	7(13.7%)	44 (86.3%)	51		
Subtype						
	Serous OC	66 (53.7%)	57 (46.3%)	123	3.629	0.304
	Endometrioid OC	6 (54.5%)	5 (45.5%)	11		
	Mucinous OC	4 (40%)	6 (60%)	10		
	Clear cell OC	6 (85.7%)	1 (14.3%)	7		
	Total	82 (54.3%)	69 (45.7%)	151		
Stage						
	I+II	28 (35.9%)	50 (64.1%)	78	22.030	<0.001
	III+IV	54 (74.0%)	19 (26.0%)	73		
	Total	82 (54.3%)	69 (45.7%)	151		

**Table 2:** Relationship between ZFP57 expression and clinical pathological parameters of patients in serous ovarian cancer.

		ZFP57 High	ZFP57 Low	Total	χ^2^	P
	Serous OC	66 (53.7%)	57 (46.3%)	123	23.607	<0.001
	ovarian cyst	7 (13.7%)	44 (86.3%)	51		
Age						
	≤50	19 (50.0%)	19 (50.0%)	38	0.296	0.586
	>50	47 (55.3%)	38 (44.7%)	85		
Grade						
	high-grade serous	56 (65.1%)	30 (34.9%)	86	13.062	<0.001
	low-grade serous	11 (29.7%)	26 (70.3%)	37		
Stage						
	I	11 (28.9%)	27 (71.1%)	38	21.147	<0.001
	II	7 (36.8%)	12 (63.2%)	19		
	III	37 (72.5%)	14 (27.5%)	51		
	IV	11 (73.3%)	4 (26.7%)	15		
	Total	66 (53.7%)	57 (46.3%)	123		
